# Molecular Profiling to Guide Fertility Sparing Therapy in Early-Stage Endometrial Cancer: A EORTC Gynaecological Cancer Group Study

**DOI:** 10.3390/cancers18152439

**Published:** 2026-07-29

**Authors:** Ramon Yarza, Reyes Oliver-Pérez, Eva Oldenburger, Lawrence Kasherman, Shira Peleg Hasson, José Manuel Estrada-Lorenzo, Beatriz Álvarez-Rodríguez, Alberto Martinez de Lara, Fernanda Herrera, Judith Kroep, Ainhoa Madariaga

**Affiliations:** 1 Drug Development and Phase I Unit, START-CIOCC, University Hospital HM Sanchinarro, 28050 Madrid, Spain; alberto.martinez@startresearch.com; 2 Department of Gynaecologic Oncology, Hospital Universitario 12 de Octubre, 28041 Madrid, Spain; m.delosreyes.oliver@salud.madrid.org; 3 School of Medicine, Universidad Complutense de Madrid, 28040 Madrid, Spain; 4 Department of Radiation Oncology, University Hospital Leuven, 3000 Leuven, Belgium; e.s.a.oldenburger@lumc.nl; 5 Department of Medical Oncology, Illawarra Cancer Care Centre, Wollongong, NSW 2500, Australia; lawrence.kasherman@health.nsw.gov.au; 6 Oncology Department, Tel Aviv Sourasky Medical Center, Tel Aviv 6423906, Israel; shiraph@tlvmc.gov.il; 7 Library and Information Services, Hospital Universitario del Sureste, 28500 Arganda del Rey, Madrid, Spain; josemanuel.estrada@salud.madrid.org; 8 Department of Radiation Oncology, Hospital Universitario HM Sanchinarro, HM Hospitales, HM CIOCC Centro Integral Oncológico Clara Campal, 28050 Madrid, Spain; balvarez@hmhospitales.com; 9 Radiation Oncology Service, Department of Oncology, Lausanne University Hospital, 1011 Lausanne, Switzerland; fernanda.herrera@chuv.ch; 10 Department of Medical Oncology, Leiden University Medical Center, 2333 Leiden, The Netherlands; j.r.kroep@lumc.nl; 11 Department of Medical Oncology, 12 de Octubre University Hospital, 28041 Madrid, Spain

**Keywords:** endometrial cancer, fertility-sparing treatment, molecular classification, objective response rate

## Abstract

**Objective**: Fertility-preserving therapies are a potential option for selected patients with early-stage endometrial cancer and atypical endometrial hyperplasia/endometrial intraepithelial neoplasia. This systematic review aims to evaluate the impact of fertility-preserving strategies according to molecular subtype. **Methods**: A comprehensive search was conducted in PubMed, Cochrane Library, EMBASE, and Web of Science for English-language studies published until June 2024, investigating fertility-sparing treatments using endocrine therapy in early-stage endometrial cancer, considering molecular classification. The PRISMA checklist was followed, and the protocol was registered in PROSPERO (CRD42025649342). Data on body mass index (BMI), age, and molecular classification (POLE-mutant, non-specific molecular profile [NSMP], p53-abnormal, and mismatch repair deficient [dMMR]) were extracted. The study assessed objective response rates (ORR), pregnancy outcomes, and survival outcomes. Statistical analyses were performed using R software. **Results**: The search identified 941 articles, and 10 studies with 457 patients were selected for final analysis. Pregnancy outcomes were reported in three studies, whilst all reported ORR data. Baseline median age (dMMR 35 years, POLE-mutant 38.2 years, p53-abnormal 33.9 years, and NSMP 31.6 years) and BMI (dMMR 24.3, POLE-mutant 27.9, p53-abnormal 26.2, and NSMP 29.8) differed by molecular subtype. Aggregated full-term delivery rates from the three studies reporting pregnancy outcomes were 36.4% (4/11) for POLE-mutant, 22.1% (27/122) for NSMP, 14.3% (1/7) for p53-abnormal, and 4.0% (1/25) for dMMR tumours, but statistical comparison was not feasible due to small sample size. Overall response rate was highest in NSMP (87.4%) and POLE-mutant tumours (84.6%), while dMMR (59.3%) and p53-abnormal (58.8%) had significantly lower ORR (compared to NSMP, *p* < 0.001 and *p* = 0.03, respectively). **Conclusions**: These findings support the potential role of molecular classification in refining patient selection for fertility-sparing treatment in early-stage endometrial cancer. Pregnancy-related outcomes should be interpreted with caution given the limited available evidence.

## Simple Summary

Preserving fertility is an important treatment option for young women with early-stage endometrial cancer who wish to have children in the future. However, not all patients respond equally well, and some experience disease persistence or recurrence. Recent advances have shown that endometrial cancer can be divided into different molecular groups, which may respond differently to treatment. In this study, we reviewed all available published evidence to investigate whether these molecular groups are associated with the success of fertility-preserving treatment. We analysed data from 457 patients, included in ten studies. Women with one molecular group had the highest treatment response rates, whereas two other groups had lower response rates and were more likely to experience disease progression. Information on pregnancy outcomes was limited, high lighting the need for further research. Our findings suggest that molecular classification may help identify patients who are more likely to benefit from fertility-preserving treatment, supporting more personalised counselling and treatment decisions while maintaining a careful balance between cancer control and future reproductive goals.

## 1. Introduction

Endometrial cancer (EC) is the most common gynaecological malignancy in developed countries, with an increasing incidence among younger women of reproductive age [1]. For these patients, the standard treatment of total hysterectomy with bilateral salpingo-oophorectomy poses a significant challenge, as it eliminates the possibility of childbearing and can profoundly impact quality of life [2,3]. Consequently, fertility-sparing therapies (FST) have garnered attention as a viable option for carefully selected patients with early-stage EC [4,5].

FST typically involves progestin-based treatment, such as oral medroxyprogesterone acetate, megestrol acetate or a levonorgestrel-releasing intrauterine device to induce tumour regression [6]. A combined approach consisting of hysteroscopic tumour resection, followed by oral progestins and/or levonorgestrel-intra-uterine device, is the most effective FST both for complete response rate and live birth rate [7]. Candidates for this type of treatment are carefully selected based on stringent criteria confirmed by imaging and histological assessments, including those with low-grade, early-stage disease confined to the endometrium. Response rates to FST range from 50 to 80%, however recurrence rates of up to 40% [6], highlight the need for close monitoring through periodic endometrial sampling. Detailed counselling about potential risks including disease progression, and the need for definitive surgical treatment once childbearing is complete, needs to be considered for each patient undergoing FST [8].

For those undergoing FST, there is emerging evidence highlighting significant variation in outcomes suggesting that a complex interplay of clinical, pathological, and molecular factors can influence treatment success, but are not fully understood [9]. Recent advances in molecular profiling of EC, such as those of the Cancer Genome Atlas (TCGA), have provided new insights into tumour biology and potential treatment outcomes [10]. Through this, EC is classified into four distinct molecular subtypes—POLE ultramutated (POLEm), microsatellite instability (MSI/dMMR) hypermutated, copy-number low (CN-low/NSMP), and copy-number high (CN-high/p53abn)—which have important prognostic and predictive implications [11,12]. However, due to its complexity, the TCGA classification was not easily translatable to clinical practice. To address this, the ProMisE (Proactive Molecular Risk Classifier for Endometrial Cancer) system was developed, categorising tumours into clinically relevant molecular subtypes based on key TCGA findings. Molecular profiling has emerged as a valuable tool for prognostication and treatment planning [7,13], and has recently been incorporated into tumour staging and treatment guidelines [14].

To build upon existing evidence and practice guidelines, this systematic review and meta-analysis aims to update the available evidence on fertility-sparing treatment in young women with endometrial cancer by incorporating recently published studies and evaluating treatment response according to molecular subtype. In addition, we explored pregnancy-related outcomes across molecular subgroups where data were available.

## 2. Materials and Methods

This systematic review and meta-analysis aimed to investigate the association between the molecular subtypes and the probability of response and recurrence in patients with localised EC treated with FST. Secondary objectives included assessing demographic variables between molecular subtypes and evaluating the association between molecular classification and pregnancy-related outcomes. The study was conducted in accordance with the Preferred Reporting Items for Systematic Reviews and Meta-Analyses (PRISMA) checklist [15]. The protocol was registered in the PROSPERO database under registration ID CRD42025649342.

A comprehensive literature search was performed in PubMed, Cochrane Library, Web of Science, and EMBASE from inception until 7 June 2024, using the strategy summarised in Supplementary File S1. Prospective and retrospective studies were included if they investigated the role of FST in EC and reported data on molecular classification. Only studies that stratified patients according to molecular subtypes were eligible for full analysis. Two independent reviewers (RY and AM) screened titles and abstracts in Covidence, selected eligible studies, and resolved discrepancies by consensus together with a third reviewer (RO). Data extraction was performed by two independent reviewers (EO and RO) to ensure accuracy and reproducibility. Publication bias assessment was not performed in this study, as all included datasets were retrospective descriptive cohorts without interventional comparisons. Traditional tools such as funnel plots or Egger’s regression are designed to detect asymmetry in comparative effect estimates, which does not apply to our pooled analysis of response rates and demographic variables (age, BMI) without inferential statistics.

The extracted variables included body mass index (BMI; continuous quantitative) and age (continuous quantitative), along with their corresponding 95% confidence intervals (95% CI) or interquartile ranges (IQR) when 95% CI was not reported. Molecular classification was recorded as a categorical variable with four subtypes: POLE-mutant, non-specific molecular profile (NSMP), p53-abnormal, and mismatch repair deficient (dMMR). Response variables, including objective response rate (ORR), as well as rates of complete response (CR), partial response (PR), stable disease (SD), and progressive disease (PD), were extracted as discrete quantitative variables. CR was defined as the absence of cancer or atypical hyperplasia in follow-up endometrial samples, with histologically normal endometrial tissue. PR was defined as pathological improvement with persistence of atypical or premalignant changes. SD was defined as persistent lesions without histological improvement or progression. PD was defined as progression of complex atypical hyperplasia (CAH) to FIGO grade 1 endometrioid adenocarcinoma (EAC), or progression of CAH or FIGO 1 EAC to a higher-grade or more invasive disease, as this histologic upgrading represents clinically meaningful disease progression that usually warrants treatment modification. Studies including patients with atypical endometrial hyperplasia/endometrial intraepithelial neoplasia undergoing fertility-sparing treatment were also considered eligible when molecular classification data were available, consistent with previous systematic reviews in this field. Pregnancy outcomes were collected as the percentage of women who carried pregnancies to term. Survival outcomes, including recurrence rates, progression-free survival (PFS) and overall survival (OS), were intended for extraction.

For the pooling of BMI and age data across different molecular subtypes, a random-effects meta-analysis using the DerSimonian–Laird method was performed. Heterogeneity was assessed using Higgins’ I^2^ statistic. When studies reported IQR but not 95% CI, the latter was approximated using the Wan method [16]. For ORR and pregnancy outcomes, crude pooled proportions were calculated by summing the total number of events and dividing by the total number of patients across all included studies within each molecular subgroup. Group comparisons were performed using Fisher’s exact test, with Bonferroni correction applied for multiple testing. Results were visualised using forest plots for pooled BMI and age data, while a descriptive table was constructed for ORR and numbers of CR and PR events. A bar plot displaying pooled ORR percentages across molecular subtypes was generated, with statistical significance (after Bonferroni adjustment) indicated by connections between significant comparisons (* for *p* = 0.01–0.05, ** for *p* = 0.001–0.01, and *** for *p* < 0.001). All statistical analyses were performed using R software (version 4.4.1).

## 3. Results

### 3.1. Study Selection

**Figure 1:**
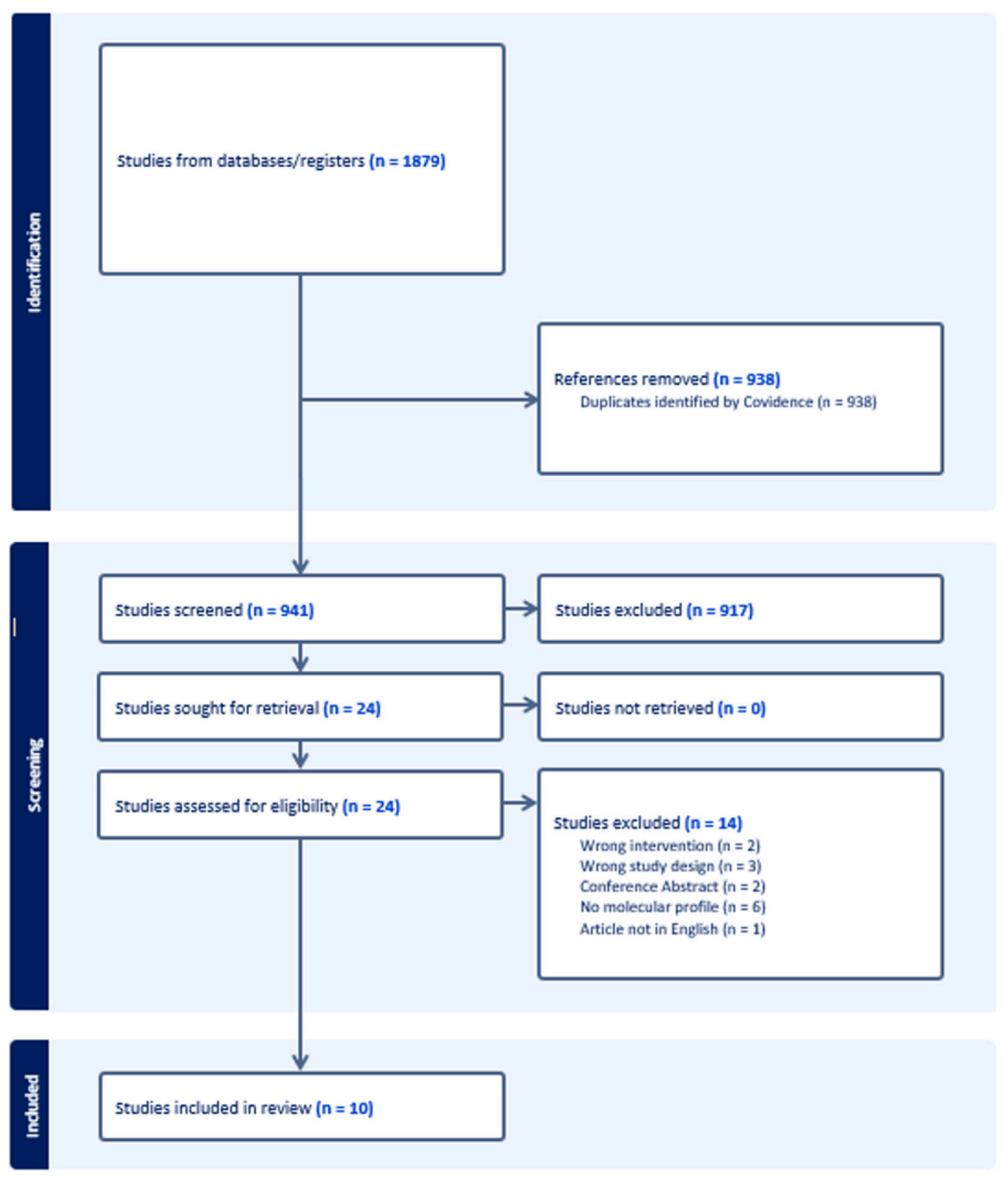
PRISMA flow diagram. PRISMA checklist see File S2.

The systematic search identified 941 articles (PRISMA flow diagram in Figure 1). After screening titles and abstracts, 24 studies were selected for full-text review, and 10 studies, summing up 457 patients, were included in the final analysis [17,18,19,20,21,22,23,24,25,26]. Among the included studies, three reported pregnancy outcomes, and all 10 reported ORR. None of the studies reported PFS or OS. All studies assessed hormonal therapy (oral, intrauterine, injected progestins, or combined approaches) as FST, and one study included hysteroscopic fertility sparing approaches with intrauterine progestins [24]. Summary descriptive statistics of all articles included are presented in Table 1.

### 3.2. Demographic Characteristics

**Figure 2:**
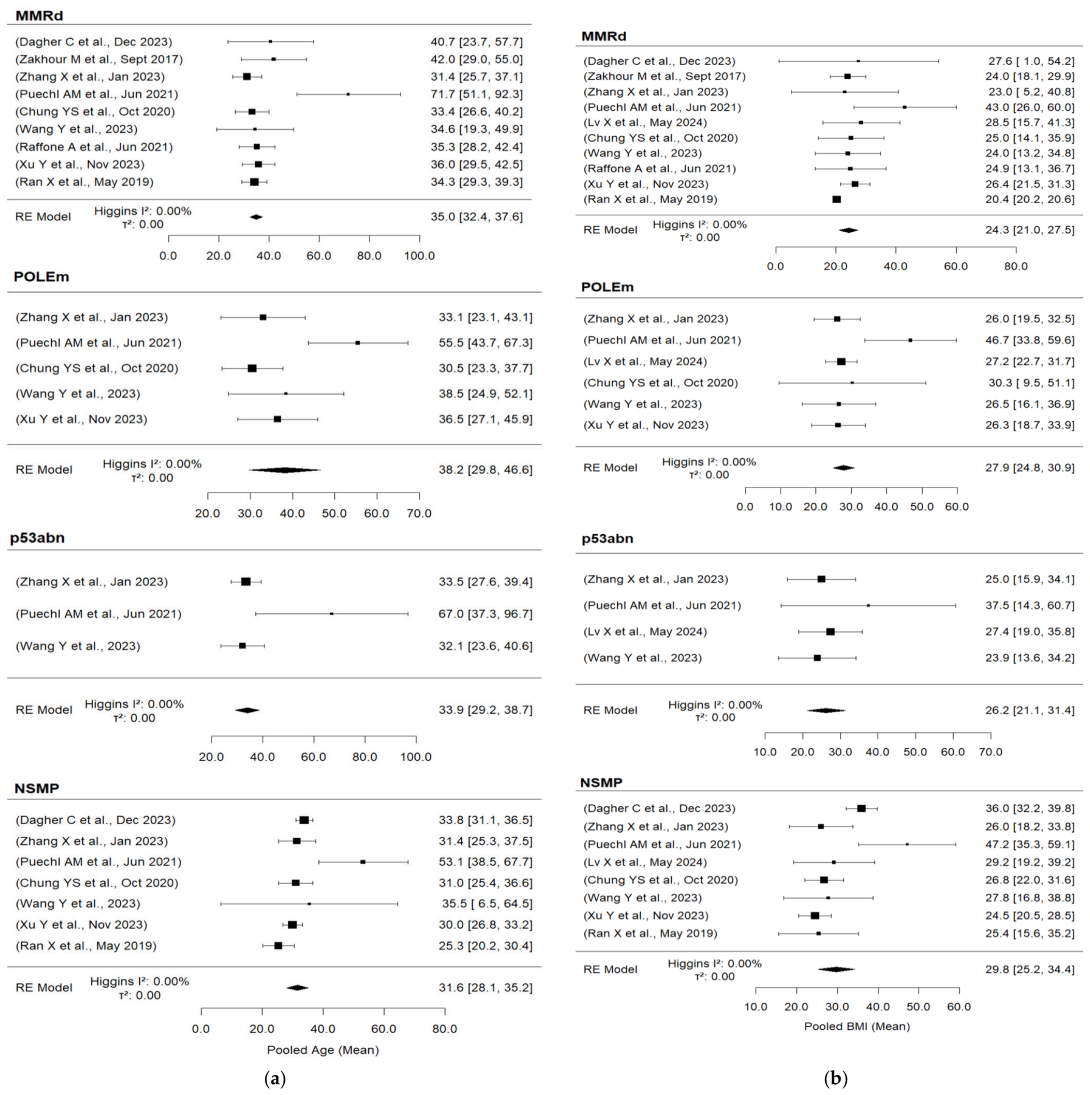
(**a**) Forest plot by age. dMMR (35 years, 95% CI: 32.4–37.6), POLE-mutant (38.2 years, 95% CI: 29.8–46.6), p53-abnormal (33.9 years, 95% CI: 29.2–38.7), and NSMP (31.6 years, 95% CI: 28.1–35.2); (**b**) Forest plot by body mass index (BMI). The pooled mean BMI for each subtype was: dMMR (24.3, 95% CI: 21.0–27.5), POLE-mutant (27.9, 95% CI: 24.8–30.9), p53-abnormal (26.2, 95% CI: 21.1–31.4), and NSMP (29.8, 95% CI: 25.2–34.4); *dMMR: mismatch repair deficient POLE mut: POLE -mutant, NSMP: non-specific molecular profile, p53abn: p53-abnormal* [17,18,19,20,21,22,23,24,25,26].

The pooled mean age varied across molecular subtypes, patients with NSMP tumours presenting the youngest mean age (31.6 years, 95% CI: 28.1–35.2), followed by p53-abnormal (33.9 years, 95% CI: 29.2–38.7), dMMR (35 years, 95% CI: 32.4–37.6), then POLE-mutant (38.2 years, 95% CI: 29.8–46.6). The meta-analysis showed no heterogeneity (Higgins’ I^2^ = 0%, τ^2^ = 0%) across all subtypes (Figure 2a).

Similarly, BMI differed among subtypes, with NSMP patients exhibiting the highest pooled mean BMI and dMMR patients the lowest. The pooled mean BMI for each subtype was: dMMR (24.3, 95% CI: 21.0–27.5), POLE-mutant (27.9, 95% CI: 24.8–30.9), p53-abnormal (26.2, 95% CI: 21.1–31.4), and NSMP (29.8, 95% CI: 25.2–34.4, Figure 2b). The meta-analysis indicated no heterogeneity (Higgins’ I^2^ = 0%, τ^2^ = 0% for all subtypes).

### 3.3. Fertility Outcomes

Three studies reported successful full-term delivery rates, and only one [17] (Lv X et al.) documented the total number of pregnancies achieved per molecular subgroup. In this paper, pregnancy attempts were reported in 93 patients with available molecular classification, with overall pregnancy success rate (defined as achieving a pregnancy, regardless of outcome) varying significantly across subtypes: 46.7% (7/15) in dMMR, 100% (6/6) in POLE-mutant, 74.6% (50/67) in NSMP, and 0% (0/5) in p53-abnormal tumours. However, full-term delivery success rates were lower, at 6.7% (1/15) in MSI-H, 50% (3/6) in POLE-mutant, 34.3% (23/67) in NSMP, and 0% (0/5) in p53-abnormal tumours.

When aggregating data from all three studies that reported full-term deliveries [17,18,19] (overall full-term delivery rates were 4.0% (1/25) for dMMR, 36.4% (4/11) for POLE-mutant, 22.1% (27/122) for NSMP, and 14.3% (1/7) for p53-abnormal tumours. Notably, while the POLE-mutant group had the highest rate of full-term deliveries, the numbers remained small, limiting statistical power for comparisons. Because pregnancy intention, pregnancy attempts, assisted reproductive technology, and complete response before conception were inconsistently reported across the included studies, further pooled analyses according to these variables were not feasible.

### 3.4. Response Rates by Molecular Subtype

**Figure 3:**
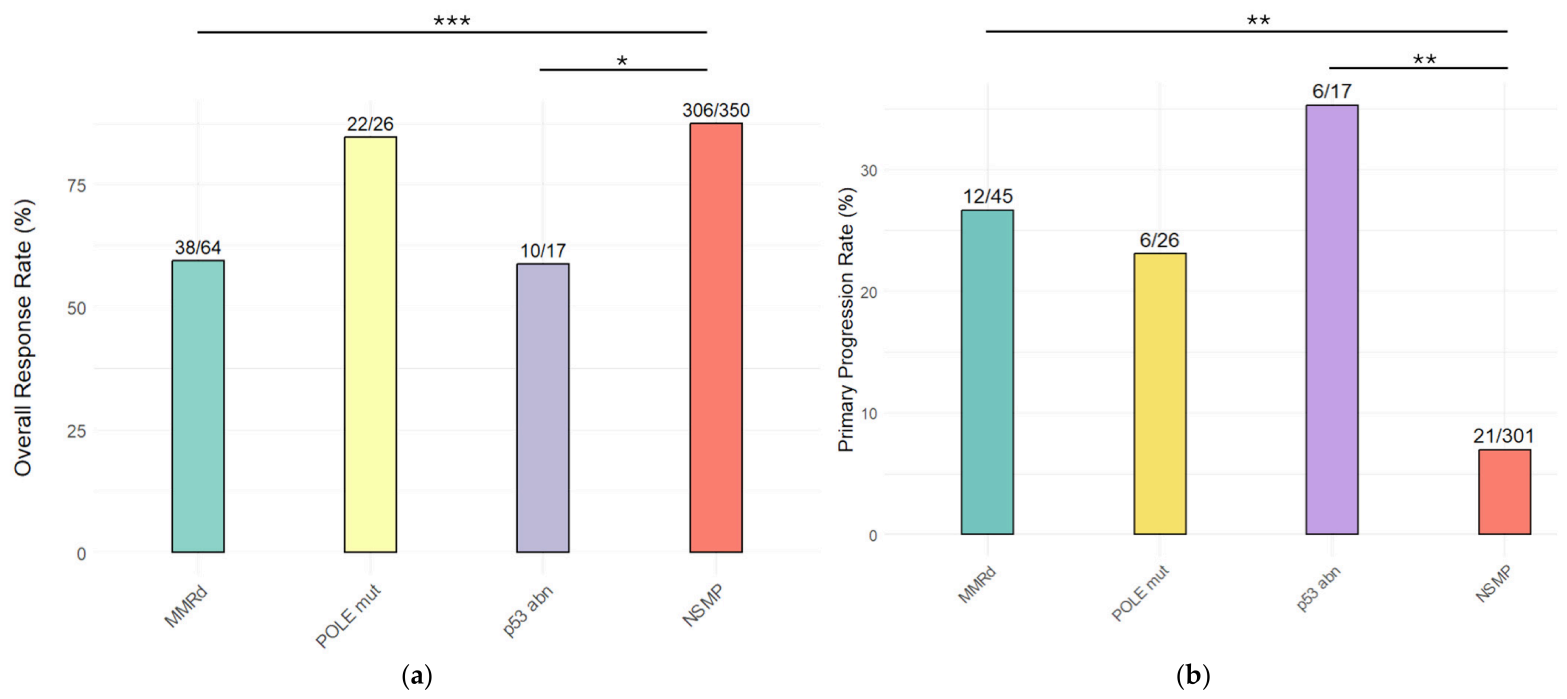
Bar plot depicting (**a**) the overall response rate and (**b**) primary progression rate (%) for molecular subtypes of endometrial cancer. Both are presented as a percentage on the y-axis, with molecular subtypes displayed on the x-axis. Each bar represents the ORR/PPR for the corresponding molecular subtype, with the numerator and denominator of responders indicated above each bar. Statistically significant differences between subtypes are denoted above the bars (* *p* < 0.05; ** = *p* < 0.01 but >0.001; *** *p* < 0.001).

Significant differences in ORR were noted across molecular subtypes (*p* < 0.001, Table 1). Patients with NSMP tumours exhibited the highest ORR (87.4%, CI 95% 83–91), followed by POLE-mutant tumours (84.6%, CI 95% 66–95), while dMMR and p53-abnormal subtypes demonstrated significantly lower ORR compared to NSMP tumours, at 59.3% (95% CI 39–78) and 58.8% (95% CI 33–82), respectively (Figure 3a). The pairwise comparisons heatmap highlights that dMMR and p53-abnormal subtypes had a statistically significant lower ORR compared to NSMP, with adjusted *p*-values < 0.001 and 0.03, respectively. Comparisons between other subtypes did not reach statistical significance after Bonferroni correction.

The primary progression rates by molecular subgroup were 7% (21/301) in NSMP, 23.1% (6/26) in POLEm, 26.7% (12/45) in MMRd and 35.3% (6/17) p53-abnormalThe pairwise comparisons heatmap showed that NSMP tumours had significantly lower primary progressions than the MMRd and p53-abnormal subgroup, with adjusted *p*-values of 0.002 and 0.008, respectively. Comparisons with the POLEm subtype did not reach statistical significance after Bonferroni correction (Figure 3b).

## 4. Discussion

This systematic review and meta-analysis focused on the impact of molecular subtyping in FST for 457 patients with EC, and to our knowledge is the largest pooled analysis to date assessing ORR and pregnancy outcomes.

The current standard management for early-stage EC is total hysterectomy, which is not a viable option for those wishing to preserve fertility. Supported by guidelines from international gynaecologic oncology societies [7], non-surgical FST options can be considered for those who meet certain criteria for low-risk disease, with variations in treatment based on timing of planned conception, in conjunction with frequent biopsy surveillance. Prior studies have demonstrated pregnancy rates after FST up to 60% and were even higher when combined with hysteroscopic resection [27,28], although these rates could be confounded by other underlying comorbidities affecting fertility, including obesity, polycystic ovarian syndrome, or type 2 diabetes [29,30,31]. In our literature search, only three out of ten studies focused on fertility outcomes preventing meaningful statistical comparisons and data interpretation. These knowledge gaps could be of potential significance in understanding which molecular subgroups are best suited to FST in terms of pregnancy outcomes, not just from the perspective of ORR and disease recurrence.

Whilst all included studies reported response rates as the outcome of interest, the assessment of ORR in this context is prone to variability due to factors such as timing of biopsies and tissue exposure to systemic therapy prior to molecular profiling and hormone receptor positivity. Due to issues with fixation that may compromise the quality of DNA for genomic analysis [32], international guidelines recommend that tissue molecular profiling should be done on biopsy tissue obtained at time of diagnosis. This will not only allow for lower DNA artifact and quality control failure rates, but if performed in a timely fashion may also assist clinicians in guiding decision making with regards to FST compared with hysterectomy, pending further prospective evidence.

In the context of recurrent or advanced EC, hormonal therapies have been used as a standard treatment option for decades, with documented response rates of up to 30% [33] for single-agent progestogens, primarily in those with hormone-receptor positive and grade 1 disease. Since the introduction of TCGA molecular classification for EC, studies have demonstrated that ER/PR-positive cases are present across all subgroups, although especially in NSMP subgroup, which explains the higher ORR. ER status within the NSMP subgroup could potentially indicate a lower-risk population [34,35]. Only one prior meta-analysis has been published previously focused on FST in early-stage EC, demonstrating that in 363 patients across eight studies there was significantly lower ORR and higher recurrence rates in dMMR and p53 abnormal subgroups compared with the NSMP subgroup [36]. Compared with the previous meta-analysis, our study incorporates two additional studies and a larger pooled population, while also providing an exploratory synthesis of pregnancy-related outcomes according to molecular subtype. These findings are consistent with prior meta-analysis, further suggesting that tumour molecular subtype may impact counselling and therapeutic decision making in this context.

In our study, no significant differences were detected across molecular subgroups by BMI, but this was highest (29.8) in the NSMP subgroup, compared with the dMMR subgroup where BMI was within the normal range (24.3). Obesity is known to be strongly associated with EC, with the worldwide increase in EC incidence thought to be due to the obesity epidemic in developed nations. Mechanistically, this is thought to be due to increased oestrogen exposure in uterine tissues, insulin resistance, and the proinflammatory state [37]. In addition to a positive correlation with carcinogenesis, obesity is also known to be associated with lower fertility rates due to metabolic syndrome or chronic ovulation disorders, thus highlighting the complexities of managing young, obese women with multimorbidity and EC. Interestingly, given that higher BMI is also associated with longer duration of treatment and higher EC recurrence rates [31], future works should not only focus on survivorship issues such as managing chronic diseases, but also consider the importance of assessing oncologic outcomes including recurrence rates and overall survival, through robustly designed prospective trials.

Although relatively less common, POLE-mutant EC is an important molecular subgroup, as these tumours are associated with more favourable survival outcomes in early-stage EC [38]. In our study, given the relatively small sample size (n = 26, 6%), assessments are limited; however ORR was 84.6% without significant variation by age or BMI, and limited data were available with regards to pregnancy outcomes. For those who have undergone standard surgical treatment for early-stage EC, prospective studies are underway to determine whether de-escalation of adjuvant therapy in POLE-mutant EC affects long-term oncologic outcomes [39], which could also be explored in EC populations considering FST to guide treatment choices.

This review has several limitations. First, all included studies were retrospective, which inherently introduces selection bias and limits the ability to establish causal relationships. Additionally, significant heterogeneity in study design, patient populations, and outcome reporting makes it difficult to perform direct comparisons across studies. Although all studies ultimately classified patients according to the four established molecular subtypes, heterogeneity in the methods used for molecular classification, including differences in testing approaches and classifier implementation, may have introduced additional variability across studies and should be considered when interpreting the pooled results. In particular, some included cohorts enrolled patients outside the typical reproductive age range, which may reduce the representativeness of the pooled outcomes for women considering fertility preservation. Sample sizes were generally small, particularly for less common molecular subtypes such as POLE-mutant and p53-abnormal tumours, which may be underrepresented and limit generalisability. Furthermore, as this was a study-level meta-analysis, we were unable to perform regression analyses to identify potential confounders or significant interactions between variables. As such, predictors of response, progression, and recurrence remain somewhat unclear, and these knowledge gaps could not be addressed through our study. This issue is particularly relevant in evaluating pregnancy outcomes where published data was scarce, preventing meaningful inferences regarding impact of molecular classification on fertility outcomes. Similarly, differences in reporting of patient characteristics, including age, BMI, baseline ovarian reserve, and comorbidities, limit our ability to interpret molecular classification as an independent predictive factor for a successful term pregnancy. Furthermore, most studies did not provide sufficient or standardised information on other clinical or pathological factors, which are also known to influence outcomes [40,41]. This prevented us from performing further analyses combining molecular, clinical, and pathological predictors of response. In addition, the heterogeneity of fertility-sparing treatment regimens and the lack of treatment-specific outcome reporting precluded subgroup analyses according to the type of hormonal therapy administered. Future prospective studies should therefore incorporate both molecular classification and hormone receptor profiling to optimise patient selection and prediction of treatment response.

Despite these limitations, this study has several strengths. As the largest pooled analysis conducted to date, this study provides a comprehensive dataset for evaluation, allowing for more robust assessments of ORR in patients treated with FST with localised EC and identification of differences between molecular subgroups. Additionally, despite inherent limitations in retrospective studies, the inclusion of multiple independent cohorts enhances the validity of our findings which may inform patient selection and individualise treatment strategies. Compared with the previous meta-analysis by Ferrari et al. [36,42], our study provides an updated and larger pooled dataset, applies statistical subgroup comparisons, and uniquely emphasises reproductive outcomes, which are critical for counselling in this setting.

Whilst molecular classifications have been established as a prognostic and predictive tool to guide therapeutic decision making in EC, its role in those who wish to preserve fertility has become clearer, by the findings in this study.

The lower ORR and higher recurrence rates in dMMR and p53abn subgroups compared with the NSMP subgroup, suggest that tumour molecular subtype may impact counselling and therapeutic decision making.

Confounding factors such as obesity and metabolic syndrome will need to be accounted for in future studies, ensuring that sufficiently prolonged follow-up periods can be used to assess oncological and pregnancy-related outcomes to safely counsel patients.

## 5. Conclusions

In summary, this updated systematic review supports an association between molecular subtype and response to fertility-sparing treatment. Molecular classification may contribute to improved patient selection; however, pregnancy-related findings remain limited and should be considered exploratory until confirmed in larger prospective studies.

## Figures and Tables

**Table 1:** Objective response rate (ORR) by molecular subtype. Summary table with the 10 articles included in the final analysis, determining complete and partial response in each molecular subgroup, showing a significant difference in ORR (*p* < 0.001). *ORR: overall response rate. dMMR: mismatch repair deficient POLE mut: POLE -mutant, NSMP: non-specific molecular profile, p53abn: p53-abnormal*.

	dMMR	POLEm	p53abn	NSMP	*p* Value *
**Dagher C et al., December 2023** [18] **(N = 20)**	3/20	1/20		16/20	
Complete response % (n/N)	0% (0/3)	0% (0/1)	-	62.5% (10/16)	
Partial response % (n/N)	0% (0/3)	0% (0/1)	-	0% (0/16)	
ORR % (n/N)	0% (0/3)	0% (0/1)		62.5% (10/16)	-
**Zakhour M et al., September 2017** [20] **(N = 6)**	6/6				
Complete response % (n/N)	0% (0/6)	-	-	-	
Partial response % (n/N)	0% (0/6)	-	-	-	
ORR % (n/N)	0% (0/6)				-
**Zhang X et al., January 2022** [21] **(N = 59)**	4/59	3/59	3/59	49/59	
Complete response % (n/N)	100% (4/4)	100% (3/3)	33% (1/3)	63.3% (31/49)	
Partial Response % (n/N)	0% (0/4)	0% (0/3)	0% (0/3)	22.4% (11/49)	
ORR % (n/N)	100% (4/4)	100% (3/3)	33% (1/3)	85.7% (42/49)	-
**Puechl AM et al., June 2021** [22] **(N = 58)**	6/58	4/58	4/58	44/58	
Complete response % (n/N)	66.6% (4/6)	75% (3/4)	50% (2/4)	86.3% (38/44)	
Partial response % (n/N)	0% (0/6)	0% (0/4)	0% (0/4)	0% (0/44)	
ORR % (n/N)	66.6% (4/6)	75% (3/4)	50% (2/4)	86.3% (38/44)	-
**Lv X et al., May 2023** [17] **(N = 93)**	15/93	6/93	5/93	67/93	
Complete response % (n/N)	73.3% (11/15)	100% (6/6)	40% (2/5)	85.1% (57/67)	
Partial response % (n/N)	0% (0/15)	0% (0/6)	20% (1/5)	6% (4/67)	
ORR % (n/N)	73.3% (11/15)	100% (6/6)	60% (3/5)	91.1% (61/67)	-
**Chung YS et al., October 2021** [23] **(N = 57)**	9/57	2/57	1/57	45/57	
Complete response % (n/N)	11.1% (1/9)	50% (1/2)	100% (1/1)	88.9% (40/45)	
Partial response % (n/N)	22.2% (2/9)	0% (0/2)	0% (0/1)	0% (0/45)	
ORR % (n/N)	33.3% (3/9)	50% (1/2)	100% (1/1)	88.9% (40/45)	-
**Wang Y et al., 2023** [19] **(N = 52)**	7/52	4/52	2/52	39/52	
Complete response % (n/N)	42.9% (3/7)	75% (3/4)	100% (2/2)	87.2% (34/39)	
Partial response % (n/N)	0% (0/7)	25% (1/4)	0% (0/2)	0% (0/39)	
ORR % (n/N)	42.9% (3/7)	100% (4/4)	100% (2/2)	87.2% (34/39)	-
**Raffone A et al., June 2021** [24] **(N = 6)**	6/6				
Complete response % (n/N)	66.6% (4/6)	-	-	-	
Partial response % (n/N)	33.3% (2/6)	-	-	-	
ORR % (n/N)	100% (6/6)	-	-	-	-
**Xu Y et al., November 2023** [25] **(N = 94)**	5/94	6/94	1/94	82/94	
Complete response % (n/N)	100% (5/5)	83.3% (5/6)	0% (0/1)	91.4% (75/82)	
Partial response % (n/N)	0% (0/5)	0% (0/6)	100% (1/1)	0% (0/82)	
ORR % (n/N)	100% (5/5)	83.3% (5/6)	100% (1/1)	91.4% (75/82)	-
**Ran X et al., May 2022** [26] **(N = 12)**	3/12		1/12	8/12	
Complete response % (n/N)	66.6% (2/3)	-	0% (0/1)	75% (6/8)	
Partial response % (n/N)	0% (0/3)	-	0% (0/1)	0% (0/8)	
ORR % (n/N)	66.6% (2/3)	-	0% (0/1)	75% (6/8)	-
**Overall (N = 457)**	**64/457**	**26/457**	**17/457**	**350/457**	
**Complete response % (n/N)**	**53% (34/64)**	**80.8% (21/26)**	**47% (8/17)**	**83.1% (291/350)**	
**Partial response % (n/N)**	**6.3% (4/64)**	**3.8% (1/26)**	**11.8% (2/17)**	**4.3% (15/350)**	
**ORR % (n/N)**	**59.3% (38/64)**	**84.6% (22/26)**	**58.8% (10/17)**	**87.4% (306/350)**	**<0.001**

* *p*-value calculated using Fisher’s exact test for comparison of proportions; -: separation.

## Data Availability

The data presented in this study are available on request from the corresponding author.

## Supplementary Materials

The following supporting information can be downloaded at: https://www.mdpi.com/article/10.3390/cancers18152439/s1, File S1: Search strategy at Pubmed, EMBASE, Cochrane Library and Web of Science; File S2: PRISMA checklist.

## References

[1] Bray F., Laversanne M., Sung H., Ferlay J., Siegel R.L., Soerjomataram I., Jemal A. (2024). Global cancer statistics 2022: GLOBOCAN estimates of incidence and mortality worldwide for 36 cancers in 185 countries. CA Cancer J. Clin..

[2] Jordan S.J., Na R., Weiderpass E., Adami H., Anderson K.E., Brandt P.A.v.D., Brinton L.A., Chen C., Cook L.S., Doherty J.A. (2021). Pregnancy Outcomes and Risk of Endometrial Cancer: A Pooled Analysis of Individual Participant Data in the Epidemiology of Endometrial Cancer Consortium. Int. J. Cancer.

[3] Amant F., Moerman P., Neven P., Timmerman D., Van Limbergen E., Vergote I. (2005). Endometrial cancer. Lancet.

[4] Jadoul P., Donnez J. (2003). Conservative treatment may be beneficial for young women with atypical endometrial hyperplasia or endometrial adenocarcinoma. Fertil. Steril..

[5] Knez J., Al Mahdawi L., Takač I., Sobočan M. (2021). The Perspectives of Fertility Preservation in Women with Endometrial Cancer. Cancers.

[6] Park J.Y., Nam J.H. (2015). Progestins in the Fertility-Sparing Treatment and Retreatment of Patients with Primary and Recurrent Endometrial Cancer. Oncologist.

[7] Rodolakis A., Scambia G., Planchamp F., Acien M., Sardo A.D.S., Farrugia M., Grynberg M., Pakiz M., Pavlakis K., Vermeulen N. (2023). ESGO/ESHRE/ESGE Guidelines for the fertility-sparing treatment of patients with endometrial carcinoma. Int. J. Gynecol. Cancer.

[8] Terzic M., Norton M., Terzic S., Bapayeva G., Aimagambetova G. (2020). Fertility preservation in endometrial cancer patients: Options, challenges and perspectives. Ecancermedicalscience.

[9] Maggiore U.L.R., Khamisy-Farah R., Bragazzi N.L., Bogani G., Martinelli F., Lopez S., Chiappa V., Signorelli M., Ditto A., Raspagliesi F. (2021). Fertility-Sparing Treatment of Patients with Endometrial Cancer: A Review of the Literature. J. Clin. Med..

[10] Talhouk A., McConechy M.K., Leung S., Yang W., Lum A., Senz J., Boyd N., Pike J., Anglesio M., Kwon J.S. (2017). Confirmation of ProMisE: A simple, genomics-based clinical classifier for endometrial cancer. Cancer.

[11] Levine D.A., The Cancer Genome Atlas Research Network (2013). Integrated genomic characterization of endometrial carcinoma. Nature.

[12] León-Castillo A., de Boer S.M., Powell M.E., Mileshkin L.R., Mackay H.J., Leary A., Nijman H.W., Singh N., Pollock P.M., Bessette P. (2020). Molecular Classification of the PORTEC-3 Trial for High-Risk Endometrial Cancer: Impact on Prognosis and Benefit from Adjuvant Therapy. TransPORTEC consortium. J. Clin. Oncol..

[13] Kommoss S., McConechy M.K., Kommoss F., Leung S., Bunz A., Magrill J., Britton H., Grevenkamp F., Karnezis A., Yang W. (2018). Final validation of the ProMisE molecular classifier for endometrial carcinoma in a large population-based case series. Ann. Oncol..

[14] Oaknin A., Bosse T., Creutzberg C., Giornelli G., Harter P., Joly F., Lorusso D., Marth C., Makker V., Mirza M. (2022). Endometrial cancer: ESMO Clinical Practice Guideline for diagnosis, treatment and follow-up. Ann. Oncol..

[15] Page M.J., McKenzie J.E., Bossuyt P.M., Boutron I., Hoffmann T.C., Mulrow C.D., Shamseer L., Tetzlaff J.M., Akl E.A., Brennan S.E. (2021). The PRISMA 2020 statement: An updated guideline for reporting systematic reviews. BMJ.

[16] Wan X., Wang W., Liu J., Tong T. (2014). Estimating the sample mean and standard deviation from the sample size, median, range and/or interquartile range. BMC Med. Res. Methodol..

[17] Lv X., Guo L., Wang C. (2023). Efficacy of fertility-sparing treatment with LNG-IUS is associated with different ProMisE subtypes of endometrial carcinoma or atypical endometrial hyperplasia. J. Gynecol. Oncol..

[18] Dagher C., Manning-Geist B., Ellenson L.H., Weigelt B., Rios-Doria E., Barry D., Abu-Rustum N.R., Leitao M.M., Mueller J.J. (2023). Molecular subtyping in endometrial cancer: A promising strategy to guide fertility preservation. Gynecol. Oncol..

[19] Wang Y., Kang N., Li L., Wang Z., Zhou R., Shen D., Wang J. (2023). Characteristics of molecular classification in 52 endometrial cancer and atypical hyperplasia patients receiving fertility-sparing treatment. Gynecol. Obstet. Clin. Med..

[20] Zakhour M., Cohen J., Gibson A., Walts A., Karimian B., Baltayan A., Aoyama C., Garcia L., Dhaliwal S., Elashoff D. (2017). Abnormal mismatch repair and other clinicopathologic predictors of poor response to progestin treatment in young women with endometrial complex atypical hyperplasia and well-differentiated endometrial adenocarcinoma: A consecutive case series. BJOG Int. J. Obstet. Gynaecol..

[21] Zhang X., Chen D., Zhao X., Wang C., He Y., Chen Y., Wang J., Shen D. (2022). Application of molecular classification to guiding fertility-sparing therapy for patients with endometrial cancer or endometrial intraepithelial neoplasia. Pathol. Res. Pract..

[22] Puechl A.M., Spinosa D., Berchuck A., Secord A.A., Drury K.E., Broadwater G., Wong J., Whitaker R., Devos N., Corcoran D.L. (2021). Molecular Classification to Prognosticate Response in Medically Managed Endometrial Cancers and Endometrial Intraepithelial Neoplasia. Cancers.

[23] Chung Y.S., Woo H.Y., Lee J.Y., Park E., Nam E.J., Kim S., Kim S.W., Kim Y.T. (2021). Mismatch repair status influences response to fertility-sparing treatment of endometrial cancer. Am. J. Obstet. Gynecol..

[24] Raffone A., Catena U., Travaglino A., Masciullo V., Spadola S., Della Corte L., Piermattei A., Insabato L., Zannoni G.F., Scambia G. (2021). Mismatch repair-deficiency specifically predicts recurrence of atypical endometrial hyperplasia and early endometrial carcinoma after conservative treatment: A multi-center study. Gynecol. Oncol..

[25] Xu Y., Zhao M., Zhang L., Wang T., Wang B., Xue Y., Xu Z., Shao W., Chen X., Wang C. (2023). Outcomes of fertility preservation treatments in patients with endometrial cancer with different molecular classifications based on an NGS panel. Front. Oncol..

[26] Ran X., Hu T., Li Z. (2022). Molecular Classification in Patients with Endometrial Cancer After Fertility-Preserving Treatment: Application of ProMisE Classifier and Combination of Prognostic Evidence. Front. Oncol..

[27] Garzon S., Uccella S., Zorzato P.C., Bosco M., Franchi M., Student V., Mariani A. (2021). Fertility-sparing management for endometrial cancer: Review of literature. Minerva Med..

[28] Zhang Z., Huang H., Feng F., Wang J., Cheng N. (2019). A pilot study of gonadotropin-releasing hormone agonist combined with aromatase inhibitor as fertility-sparing treatment in obese patients with endometrial cancer. J. Gynecol. Oncol..

[29] Gallos I.D., Yap J., Rajkhowa M., Luesley D.M., Coomarasamy A., Gupta J.K. (2012). Regression, relapse, and live birth rates with fertility-sparing therapy for endometrial cancer and atypical complex endometrial hyperplasia: A systematic review and meta-analysis. Am. J. Obstet. Gynecol..

[30] Chao A.S., Chao A., Wang C.J., Lai C.H., Wang H.S. (2011). Obstetric outcomes of pregnancy after conservative treatment of endometrial cancer: Case series and literature review. Taiwan. J. Obstet. Gynecol..

[31] Yang B., Xie L., Zhang H., Zhu Q., Du Y., Luo X., Chen X. (2018). Insulin resistance and overweight prolonged fertility-sparing treatment duration in endometrial atypical hyperplasia patients. J. Gynecol. Oncol..

[32] Berrino E., Bellomo S.E., Chesta A., Detillo P., Bragoni A., Gagliardi A., Naccarati A., Cereda M., Witel G., Sapino A. (2024). Alternative Tissue Fixation Protocols Dramatically Reduce the Impact of DNA Artifacts, Unraveling the Interpretation of Clinical Comprehensive Genomic Profiling. Lab. Investig..

[33] Pijnenborg J.M., van Weelden W.J., Reijnen C., Xanthoulea S., Romano A. (2024). Redefining the Position of Hormonal Therapy in Endometrial Cancer in the Era of Molecular Classification. JCO.

[34] Jamieson A., Singh N., Huvila J., Gilks C.B., McAlpine J.N. (2023). The continuing evolution of endometrial carcinoma molecular classification: Risk stratification within the No Specific Molecular Profile (NSMP) subtype. Gynecol. Oncol..

[35] Vermij L., Jobsen J.J., León-Castillo A., Brinkhuis M., Roothaan S., Powell M.E., de Boer S.M., Khaw P., Mileshkin L.R., TransPORTEC Consortium (2023). Prognostic refinement of NSMP high-risk endometrial cancers using oestrogen receptor immunohistochemistry. Br. J. Cancer.

[36] Ferrari F.A., Uccella S., Franchi M., Scambia G., Fanfani F., Fagotti A., Pavone M., Raspagliesi F., Bogani G. (2025). Performance of molecular classification in predicting oncologic outcomes of fertility-sparing treatment for atypical endometrial hyperplasia and endometrial cancer. Int. J. Gynecol. Cancer.

[37] Crosbie E.J., Kitson S.J., McAlpine J.N., Mukhopadhyay A., Powell M.E., Singh N. (2022). Endometrial cancer. Lancet.

[38] De Boer S.M., Powell M.E., Mileshkin L., Katsaros D., Bessette P., Haie-Meder C., Ottevanger P.B., Ledermann J.A., Khaw P., Colombo A. (2018). Adjuvant chemoradiotherapy versus radiotherapy alone for women with high-risk endometrial cancer (PORTEC-3): Final results of an international, open-label, multicentre, randomised, phase 3 trial. Lancet Oncol..

[39] RAINBO Research Consortium (2023). Refining adjuvant treatment in endometrial cancer based on molecular features: The RAINBO clinical trial program. Int. J. Gynecol. Cancer.

[40] Mirza M.R., Bjørge L., Marmé F., Christensen R.D., Gil-Martin M., Auranen A., Ataseven B., Rubio M.J., Salutari V., Luczak A.A. (2025). Palbociclib plus letrozole in estrogen receptor-positive advanced/recurrent endometrial cancer: Double-blind placebo-controlled randomized phase II ENGOT-EN3/PALEO trial. Gynecol. Oncol..

[41] Zelisse H.S., Snijders M.L., Groenendijk F.H., Halfwerk J.B., Hooijer G.K., van Driel W.J., León-Castillo A., Lok C.A., Kooreman L.F., Lambrechts S. (2025). The prognostic potential of molecular subtypes including estrogen receptor status in endometrioid ovarian cancer. Gynecol. Oncol..

[42] Agusti N., Kanbergs A., Nitecki R. (2024). Potential of molecular classification to guide fertility-sparing management among young patients with endometrial cancer. Gynecol. Oncol..

