# Supplementary material for: Molecular Profiling to Guide Fertility Sparing Therapy in Early-Stage Endometrial Cancer: A EORTC Gynaecological Cancer Group Study
Source: Cancers (Basel). 2026 Jul 29;18(15):2439. doi: 10.3390/cancers18152439 (PMC13465110; doi:10.3390/cancers18152439)
Supplement: Supplementary file 1 [file cancers-18-02439-s001.zip › cancers-4392795-Supplementary File S1.pdf]

Supplementary File S1. Search strategy at Pubmed, EMBASE, Cochrane Library and Web of Science.

PubMed 10/6/2024 = 530

#1 endometr* AND (cancer OR neoplas* OR metastas* OR tumor* OR tumour* OR carcinoma)

#2 "Endometrial Neoplasms"[Mesh]

#3 #1 OR #2

#4 fertility AND sparing AND (treatment OR therap*)

#5 #3 AND #4

Embase 10/6/2024 = 560

#1 endometr* AND ('cancer'/exp OR cancer OR neoplas* OR metastas* OR tumor* OR tumour* OR 'carcinoma'/exp OR carcionoma)

#2 'endometrium tumor'/exp

#3 #1 OR #2

#4 fertility AND sparing AND (treatment OR therap*)

#5 #3 AND #4

#6 #5 AND ('article'/it OR 'article in press'/it OR 'editorial'/it OR 'letter'/it OR 'note'/it OR review'/it OR 'short survey'/it)

#7 #6 AND [embase]/lim

Cochrane Library 10/6/2024 = 39

#1 endometr* AND (cancer OR neoplas* OR metastas* OR tumor* OR tumour* OR carcinoma)

#2 MeSH descriptor: [Endometrial Neoplasms] explode all tres

#3 #1 OR #2

#4 fertility AND sparing AND (treatment OR therap*)

#5 #3 AND #4

Web of Science 10/6/2024 = 750

#1 TOPIC= endometr* AND (cancer OR neoplas* OR metastas* OR tumor* OR tumour* OR carcinoma)

#2 TOPIC= fertility AND sparing AND (treatment OR therap*)

#3 #1 AND #2

#4 #3 NOT (Meeting OR Abstracts OR Book)
